# Case Report: Acute Testicular Pain as the Initial Manifestation of a Ruptured Common Iliac Artery Aneurysm

**DOI:** 10.3390/reports9030266

**Published:** 2026-08-12

**Authors:** Cristina Valeria Tandara, Alexandru Cristian Cindrea, Adina Maria Mârza, Lucian Adrian Tandara, Ovidiu Alexandru Mederle

**Affiliations:** 1 Emergency Department, Emergency Clinical Municipal Hospital, 300254 Timisoara, Romania; cristinatandara81@gmail.com; 2 Doctoral School, Faculty of General Medicine, “Victor Babes” University of Medicine and Pharmacy Timisoara, 300041 Timisoara, Romania; 3 Centre for Artificial Intelligence and Big Data in Emergency Medicine and Clinical Research, Department of Surgery, “Victor Babes” University of Medicine and Pharmacy, 300041 Timisoara, Romania; marza.adina@umft.ro (A.M.M.); mederle.ovidiu@umft.ro (O.A.M.); 4 Department of Surgery, “Victor Babes” University of Medicine and Pharmacy, 300041 Timisoara, Romania; 5 Emergency Department, “Pius Brinzeu” Emergency Clinical County Hospital, 300736 Timisoara, Romania; adrian_tandara@yahoo.com

**Keywords:** common iliac artery aneurysm, testicular pain, hydronephrosis, retroperitoneal hematoma, case report

## Abstract

**Background and Clinical Significance:** Ruptured common iliac artery aneurysms may mimic urological disease, particularly when retroperitoneal bleeding causes ureteral compression and secondary hydronephrosis. This case is clinically relevant because the first symptom was acute stabbing testicular pain, followed by lumbar discomfort and ultrasound findings suggestive of obstructive uropathy. **Case Presentation**: A 73-year-old man with arterial hypertension and active smoking presented to the Emergency Department with sudden left lumbar pain that initially radiated to, or was perceived in, the left testicle. The testicular pain resolved spontaneously, but persistent poorly localized lumbar pain continued. He was afebrile and initially hemodynamically stable. Point-of-care ultrasound showed left hydronephrosis, while the abdominal aorta appeared unremarkable. Because the patient looked clinically unwell and the abrupt, stabbing onset was not fully explained by uncomplicated renal colic, contrast-enhanced abdominal and pelvic computed tomography was performed. CT revealed a fissured saccular aneurysm of the left common iliac artery with active contrast extravasation and a large retroperitoneal hematoma compressing the ipsilateral ureter, thereby causing secondary hydronephrosis. The patient underwent urgent endovascular treatment with percutaneous transluminal angioplasty and Bentley stent. Post-revascularization CT showed no further contrast extravasation, and the patient was discharged after seven days without complications. **Conclusions:** In older patients with cardiovascular risk factors, sudden testicular, flank, or lumbar pain may warrant consideration of retroperitoneal vascular emergencies, even when initial ultrasound suggests a urological diagnosis. In this patient, discordance between the clinical presentation and the initial ultrasound findings prompted contrast-enhanced CT, which enabled diagnosis and timely vascular management.

## 1. Introduction and Clinical Significance

Common iliac artery aneurysms are focal dilatations of the common iliac arteries that exceed 18 mm in men and 15 mm in women (about 1.5 times the expected normal vessel diameter). In older patients, the underlying pathology is most often degenerative and atherosclerotic, although pseudoaneurysm, penetrating ulcer, post-dissection aneurysm, mycotic aneurysm, trauma-related disease, and connective-tissue disorders should be considered according to the clinical context [1].

Isolated iliac artery aneurysms are uncommon. European Society for Vascular Surgery (ESVS) guidelines reports that isolated iliac artery aneurysms account for up to 7% of all aortoiliac aneurysms, are predominantly diagnosed in men, and usually occur after the age of 70 years [1]. Other reports estimate isolated common iliac artery aneurysms to represent only 0.4–1.9% of all aneurysms, with a general population incidence of approximately 0.03% [2]. Robust emergency department-specific incidence data are lacking; the available literature on emergency department (ED) presentations consists mainly of isolated case reports, small emergency repair series, and vascular registry analyses [3,4,5,6,7,8].

In the emergency department, fissured iliac aneurysms may initially resemble renal colic, testicular torsion, epididymo-orchitis, pyelonephritis, diverticulitis, lumbar radiculopathy, or nonspecific retroperitoneal pain [1,7,9,10,11].

Management depends on symptoms, rupture status, hemodynamic stability, anatomy, infection risk, and available vascular expertise. Symptomatic or fissured aneurysms, however, require urgent vascular assessment irrespective of diameter. Open repair remains necessary in unstable patients, infected fields, or unsuitable anatomy, while endovascular repair is increasingly used in selected stable patients with suitable anatomy and is associated with favorable early outcomes [1,12,13,14,15].

This case report describes a ruptured common iliac artery aneurysm presenting with transient testicular pain and secondary hydronephrosis and discusses the diagnostic challenge created by apparently supportive urological ultrasound findings.

## 2. Case Presentation

A 73-year-old man presented to the ED with acute-onset pain in the left lumbar region. The symptoms had begun approximately six hours before presentation. At onset, he described a sudden, sharp, “stabbing” pain radiating to or perceived in the left testicle. This initial testicular pain resolved spontaneously; however, it was followed by persistent, dull, poorly localized pain in the left lumbar area. The patient appeared distressed and clinically unwell, despite the absence of overt hemodynamic instability at the initial assessment.

His medical history was significant for arterial hypertension and active smoking. No previous abdominal, urological, or vascular disease was reported, and the remaining medical history was unremarkable.

On physical examination, the patient was ill appearing, with persistent left lumbar discomfort. Abdominal inspection was unremarkable, with no visible distension, pulsatile mass, skin changes, or signs of trauma. Palpation elicited mild tenderness in the left flank, particularly on deep examination, without guarding or rebound tenderness. The testes were non-tender and palpably normal. Dorsalis pedis and posterior tibial pulses were initially palpable bilaterally. The patient was afebrile, with an initial blood pressure of 130/70 mmHg and a heart rate of 100 beats per minute.

Initial point-of-care venous blood gas analysis showed no relevant metabolic abnormalities. Hemoglobin was 10.7 g/dL. Given the localization of pain and the initial clinical suspicion of a possible obstructive urological cause, point-of-care ultrasound was performed. Bedside ultrasonography demonstrated grade II–III left hydronephrosis and a left cortical renal cyst. Several small hyperechoic foci were also observed within the renal parenchyma. The abdominal aorta was evaluated during the same examination and was considered sonographically unremarkable, with no evidence of aneurysmal dilatation or free intraperitoneal fluid. Urinalysis did not show clinically significant hematuria.

Although the flank-to-testicular pain and ipsilateral hydronephrosis initially supported a working diagnosis of renal colic, this diagnosis did not adequately explain the complete clinical picture. The patient appeared disproportionately unwell despite preserved hemodynamic parameters, and the abrupt, stabbing onset followed by persistent, poorly localized lumbar pain was atypical for uncomplicated ureteric obstruction. His hemoglobin was 10.7 g/dL, with no known baseline value, and urinalysis showed no significant hematuria. Although neither mild anemia nor the absence of hematuria alone excludes ureterolithiasis, their presence alongside the patient’s age, cardiovascular risk factors, and ill appearance reduced confidence in a benign urological diagnosis. No definite obstructing ureteral calculus was identified on point-of-care ultrasound. Therefore, contrast-enhanced CT of the abdomen and pelvis was requested to evaluate an alternative retroperitoneal cause.

During the period of observation in the emergency department, the patient was reevaluated multiple times. He began to report new pain in the posterior aspect of the left calf. Repeat examination showed progressive discoloration of the left lower limb, with diminished dorsalis pedis/posterior tibial Doppler signals. This change in symptom pattern raised additional concern for evolving vascular compromise, despite the initially palpable bilateral pulses and preserved hemodynamic stability.

**Figure 1:**
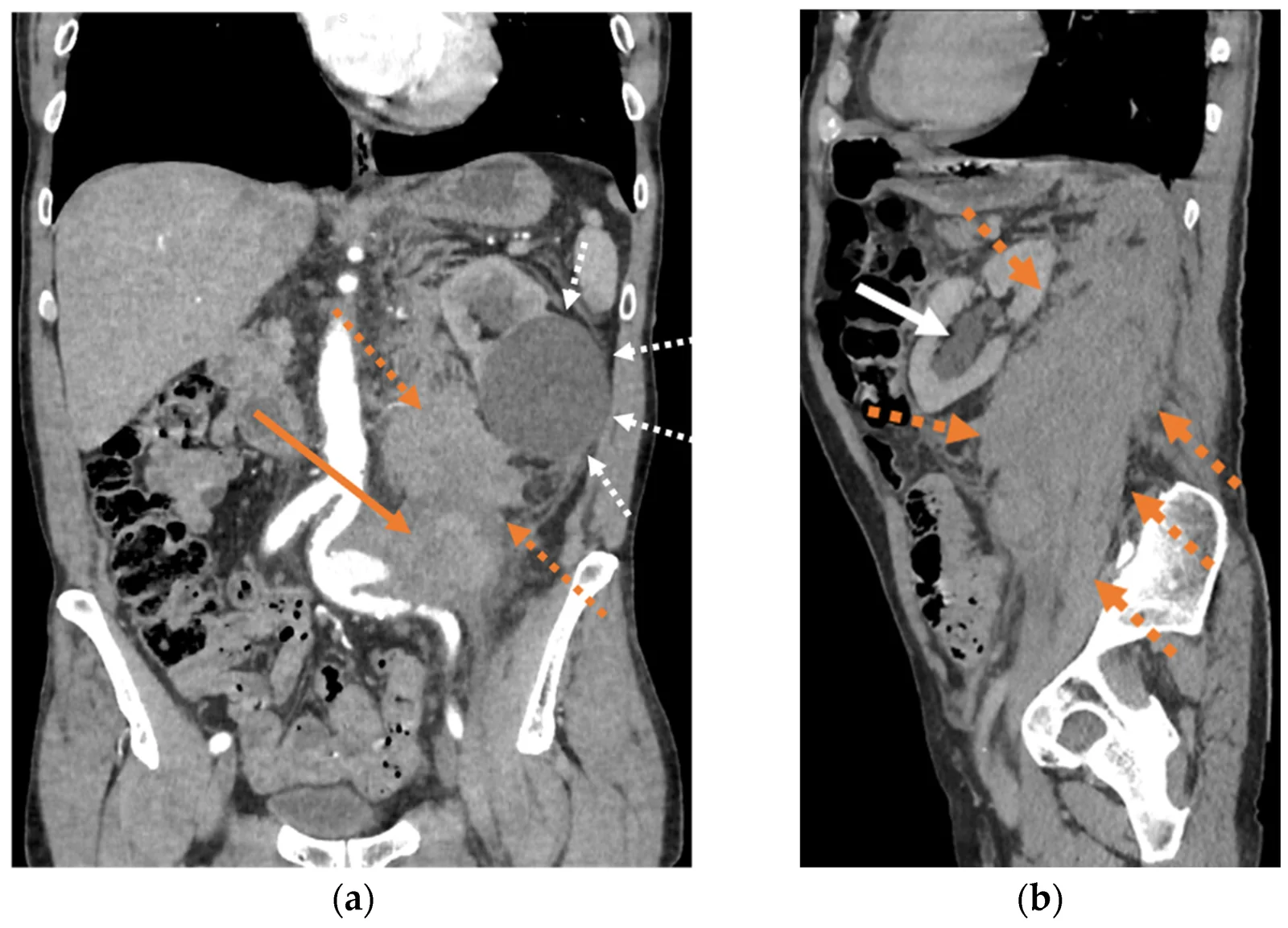
Contrast-enhanced computed tomography of the abdomen and pelvis obtained in the emergency department. (**a**) Arterial-phase coronal image showing a fissured saccular aneurysm of the left common iliac artery, measuring approximately 4.0 × 2.5 × 3.0 cm (orange arrow), with active contrast extravasation and an associated retroperitoneal hematoma measuring approximately 20 × 9 × 6.5 cm (orange dashed arrows). A large left renal cortical cyst, measuring approximately 7.3 × 6.0 × 7.0 cm, is indicated by dashed white arrows. (**b**) Delayed-phase sagittal image demonstrating the craniocaudal extent of the retroperitoneal hematoma (orange dashed arrows) and its mass effect on the left kidney and adjacent abdominal structures, resulting in grade II–III left hydronephrosis (white arrow).

Contrast-enhanced abdominal CT (Figure 1) revealed a fissured saccular aneurysm of the left common iliac artery, complicated by a retroperitoneal hematoma. Active contrast extravasation was present, indicating ongoing bleeding. The retroperitoneal hematoma was compressing the ipsilateral ureter, resulting in secondary left hydronephrosis. No primary urinary tract obstructive lesion was identified. Additional CT findings included complete thrombosis of the right internal iliac artery with distal reconstitution, multiple mural thrombi within the left internal iliac artery, and a hypodense intimal flap-like lesion involving the left external iliac artery.

**Figure 2:**
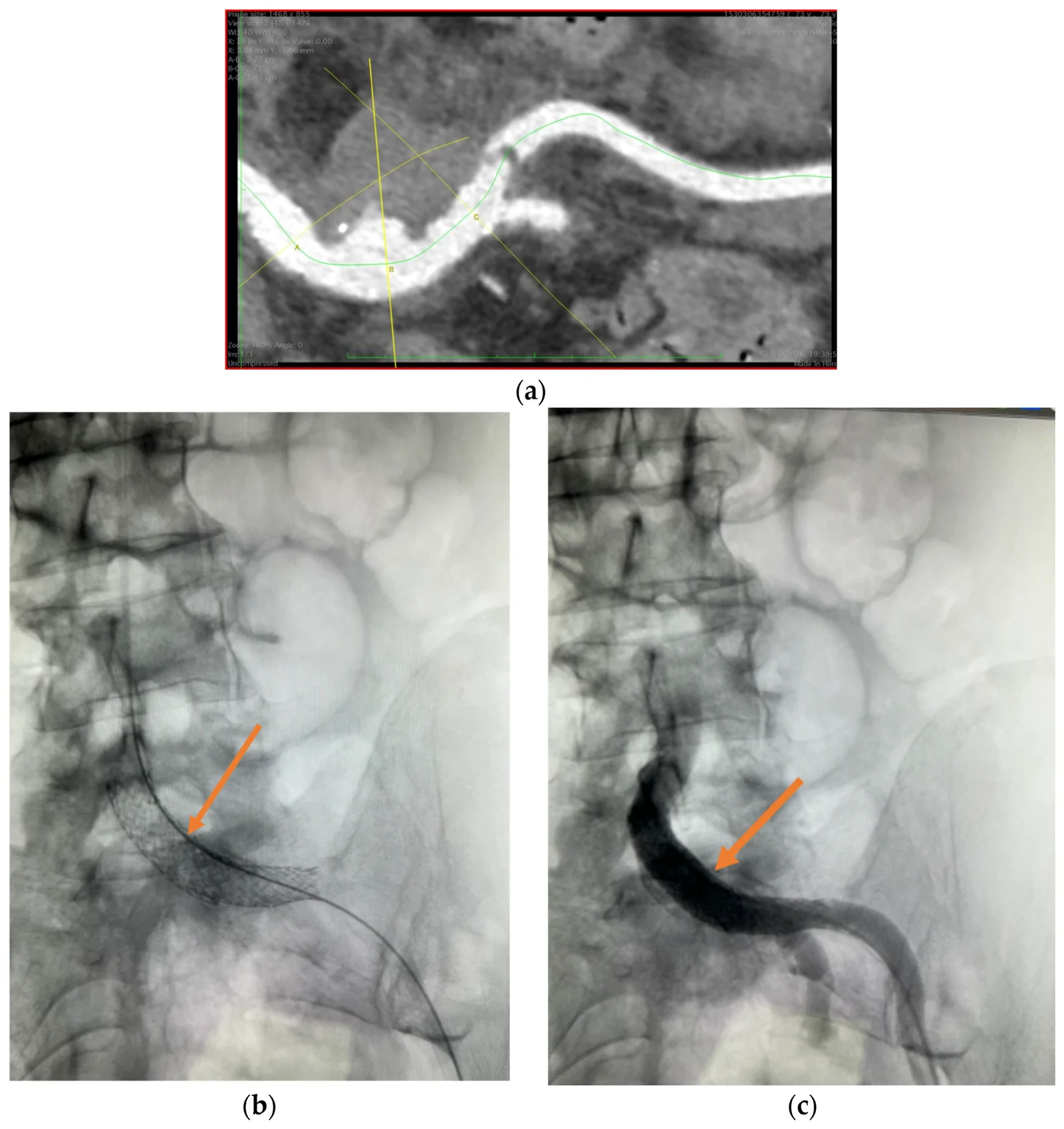
(**a**) Curved planar reformatted contrast-enhanced CT angiographic image of the left common iliac artery, demonstrating the aneurysmal arterial segment and the adjacent retroperitoneal hematoma. The green line represents the vessel centerline used to generate the curved planar reconstruction. The yellow lines indicate selected orthogonal cross-sectional planes. (**b**) Fluoroscopic image obtained during positioning and deployment of the Bentley BeGraft stent graft across the aneurysmal left common iliac artery segment (orange arrow). (**c**) Diagnostic contrast angiography performed after stent-graft deployment, demonstrating preserved flow through the treated left common iliac artery and no contrast opacification of the aneurysmal sac, consistent with successful aneurysm exclusion. The matching orange arrow indicates the treated aneurysmal segment.

Following the diagnosis, the vascular surgery team was urgently consulted, and the patient was admitted to the vascular surgery ward for definitive management. As he remained hemodynamically stable at the time of diagnosis, an endovascular approach was considered the preferred treatment strategy. Before the patient’s surgery, Doppler ultrasonography was performed, showing no anterior or tibial artery pulses. Diagnostic angiography was also performed before surgery (Figure 2). Percutaneous transluminal angioplasty (PTA) through the left femoral artery with a Bentley BeGraft aortic stent was performed. This approach was chosen because it is less invasive and allows faster recovery with fewer complications.

**Figure 3:**
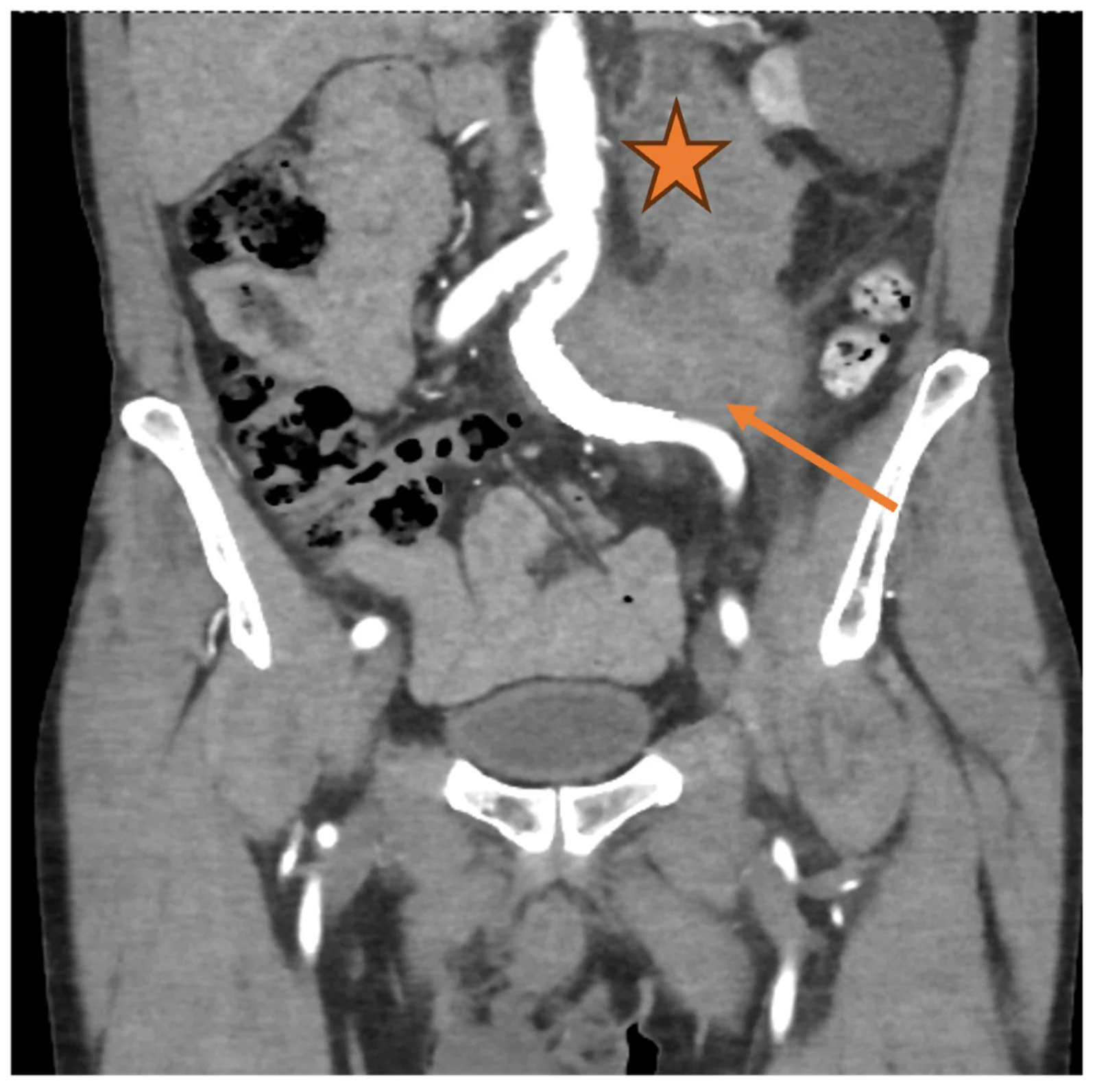
Post-revascularization angio-CT (coronal view) showing no extravasation of the contrast in the aneurysmal lumen (orange arrow) and a slight reduction in the size of the retroperitoneal hematoma (orange star).

During hospitalization, the evolution was favorable. In the second day post revascularization, the patient’s symptoms improved markedly, with complete resolution of pain and a clinically well-vascularized limb. No hematoma at the puncture site was observed. The evolution of the laboratory workup is summed up in Table 1. Before discharge, a post-revascularizing angio-CT (Figure 3) of the aorta and lower limbs was performed, showcasing no contrast extravasation and good permeability of the vessels. The patient was discharged after 7 days of hospitalization with no complications. He was scheduled for a follow-up in 2 weeks.

The diagnostic and therapeutic course, from emergency department presentation to hospital discharge, is summarized in Table 2.

The patient reported that the abrupt onset of pain was frightening and expressed gratitude for the prompt diagnosis and treatment, stating that he was relieved to have recovered without complications.

## 3. Discussion

This case highlights an unusual presentation of a fissured left common iliac artery aneurysm in a 73-year-old man, whose first symptom was sudden stabbing pain referred to the left testicle, later evolving into persistent lumbar discomfort. Although initial ultrasound suggested a urological cause through the presence of left hydronephrosis, contrast-enhanced CT revealed a fissured iliac aneurysm with retroperitoneal hematoma compressing the ureter. The case underscores the need to consider retroperitoneal vascular emergencies in older patients with cardiovascular risk factors and atypical testicular, flank, or lumbar pain, even when initial findings appear to support a benign urological diagnosis.

The diagnosis of these aneurysms in a crowded ED is difficult due to a handful of factors. Misdiagnosis of ruptured aortoiliac aneurysms—often as renal colic or musculoskeletal back pain—occurs in up to 39% of cases and is associated with increased mortality [16,17,18], with renal colic being a common misdiagnosis [19]. Their deep pelvic location means they are rarely palpable on clinical examination, and even substantial aneurysms may remain entirely asymptomatic until rupture or expansion compresses adjacent structures [20]. When they rupture, the retroperitoneal course of the bleeding—contained, at least initially, by the surrounding fascial planes—can paradoxically produce a deceptively benign external appearance, with preserved hemodynamic stability and no peritoneal signs, as was the case in our patient [16,19].

The genitofemoral nerve (L1–L2) runs along the psoas muscle and divides into a femoral branch and a genital branch; the latter crosses anteriorly to the common iliac vessels before entering the inguinal canal, where it supplies the cremaster muscle and the scrotal skin [21]. Acute hematoma formation inside the psoas muscles sheath or aneurysmal expansion along the iliac vessels can therefore directly irritate or compress the genitofemoral nerve, generating referred pain that is perceived as testicular in origin [22]. The spontaneous resolution of the testicular pain in our patient, followed by the emergence of dull lumbar discomfort, likely reflects the evolution from acute nerve irritation to sustained retroperitoneal hematoma expansion.

The masquerade performed by iliac aneurysm rupture is well documented in the literature, though the isolated testicular presentation remains uncommon enough to be the subject of individual case reports. Dolan and Zino [8] described a 56-year-old who arrived with acute testicular pain and hypotension, initially prompting a suspicion of testicular torsion, in whom CT angiography confirmed a fissured iliac aneurysm. Bray et al. [7] similarly reported a large ruptured common iliac aneurysm presenting with acute scrotal pain and bruising, noting that isolated scrotal presentations appear in only one or two prior case reports across the entire literature. Khan et al. [23] described a leaking abdominal aortic aneurysm presenting with testicular pain in a hypertensive smoker, a risk-factor profile nearly identical to our patient’s. Maharaj et al. [24] presented a 68-year-old man in whom a ruptured AAA caused severe acute testicular pain associated with syncope. These cases collectively illustrate that the referred testicular pain pathway—via genitofemoral nerve irritation—is not idiosyncratic to any single vascular lesion, but is rather a predictable consequence of retroperitoneal hematoma whenever the hematoma is anatomically proximate to the nerve’s course along the iliac vessels or psoas.

Beyond the testicular presentation, iliac and aortoiliac aneurysms are well recognized mimics of renal colic, diverticulitis, lumbar radiculopathy, pyelonephritis, appendicitis, and even cholecystitis [25,26,27,28]. The mechanism in cases presenting with urological features frequently involves ureteral compression, as retroperitoneal expansion of the aneurysm, or, as in our patient, the accompanying hematoma, extrinsically obstructs the ureter, producing secondary hydronephrosis without any intrinsic urological disease [29,30,31]. The urological findings are therefore a consequence, not a cause, yet they can convincingly anchor the diagnostic workup to the urinary tract if the overall clinical picture is not evaluated as a whole.

The later development of calf pain and limb discoloration represented an additional vascular warning sign. In the setting of iliac aneurysm rupture, this may reflect reduced iliac flow, thromboembolism, local dissection/intimal injury, or compression-related compromise. This evolution further supported urgent vascular imaging and intervention.

Bedside POCUS demonstrated grade II–III left hydronephrosis and a renal cortical cyst, while the abdominal aorta appeared sonographically normal, initially supporting a working diagnosis of ureteric obstruction. However, the abrupt stabbing onset, transient testicular pain, ill appearance despite preserved hemodynamics, anemia, absence of significant hematuria, older age, smoking history, and subsequent limb symptoms were discordant with uncomplicated renal colic and prompted contrast-enhanced CT. This distinction is important because the deep pelvic location of the iliac vessels, bowel gas, and body habitus may limit ultrasound assessment of the retroperitoneum; therefore, a normal aortic examination does not exclude iliac artery pathology, active bleeding, or retroperitoneal hematoma [32,33,34,35]. Moreover, hydronephrosis may provide a plausible but incomplete explanation and contribute to premature diagnostic closure. When POCUS findings do not adequately explain the patient’s clinical condition or evolving trajectory, cross-sectional imaging should be considered [16,17,36]. (see Table 3).

Once the diagnosis was established by CT, the management decision was relatively straightforward. The patient remained hemodynamically stable throughout, the retroperitoneal hematoma was contained, and the aneurysm anatomy was amenable to endovascular repair. Multiple series and comparative studies support endovascular repair as the preferred approach for isolated iliac artery aneurysms when anatomy and hemodynamic status permit, citing lower perioperative mortality, reduced transfusion requirements, shorter intensive care and hospital stays, and equivalent intermediate-term patency compared with open repair. In the series by Chaer et al. [41], perioperative mortality was 33% for endovascular versus 50% for open repair in ruptured isolated iliac aneurysms, with the endovascular group also showing significantly lower transfusion rates and shorter length of stay. Hechelhammer et al. [42] reported 100% assisted primary technical success rate in 11 patients undergoing emergency stent grafting for ruptured isolated iliac aneurysms, with an 18% 30-day mortality, and no late deaths or surgical conversions at a mean follow-up of 23 months. The favorable, uncomplicated seven-day hospital course in our patient was consistent with these outcomes in hemodynamically stable fissured aneurysms treated promptly by an experienced team.

The educational value of this case is primarily related to the diagnostic sequence. Transient testicular pain was followed by lumbar discomfort, while hydronephrosis on POCUS initially suggested a urological cause. The persistence of clinical features not fully explained by uncomplicated renal colic prompted CT, which demonstrated the ruptured iliac aneurysm and retroperitoneal hematoma. This case therefore provides a clinical reminder that an apparently relevant ultrasound finding may represent a secondary consequence rather than the primary cause of the patient’s symptoms.

This report has limitations. It describes a single patient, and the mechanism of testicular pain is inferred from the anatomical relationship between the retroperitoneal hematoma, iliac vessels, psoas region, and genitofemoral nerve rather than directly demonstrated. In addition, no long-term follow-up is yet available. Therefore, the case should be interpreted as a diagnostic reminder rather than evidence for a specific imaging algorithm in ED.

## 4. Conclusions

This case highlights that iliac artery aneurysm rupture may mimic urological disease, presenting with acute testicular pain and hydronephrosis from ureteral compression by retroperitoneal hematoma. In older patients with sudden testicular, flank, or lumbar pain, discordant clinical features may require urgent CT angiography to exclude vascular pathology.

## Figures and Tables

**Table 1:** Laboratory bloodwork of the patient.

Bloodwork	ED	Day 1	Day 3	Day 5
Hemoglobin [g/dL]	10.77	8.76	8.34	8.52
Hematocrit [%]	31.7	25.8	24.6	25.4
Thrombocytes [×10^3^/µL]	255.7	205.0	252.6	286.6
Creatinine [mg/dL]	1.4	1.2	1.1	1.1
Triglycerides [mg/dL]	-	66	-	-
LDL cholesterol [mg/dL]	-	76	-	-
HDL cholesterol [mg/dL]	-	31.5	-	-
Cholesterol [mg/dL]	-	112	-	-
Urine RBCs [/hpf]	1.6	-	-	-

Abbreviations: ED—emergency department; RBCs—red blood cells.

**Table 2:** Timeline of clinical presentation, diagnosis, and management.

Timeline	Main Events
0–30 min	Emergency department arrival Initial assessment—left lumbar pain preceded by sudden, stabbing left testicular pain. Patient clinically unwell but hemodynamically stable. Testicular examination was normal, and distal lower limb pulses were initially palpable.
30–60 min	Hemoglobin of 10.7 g/dL and no significant hematuria. POCUS—grade II–III left hydronephrosis and a renal cortical cyst; the abdominal aorta appeared sonographically normal.
1–2 h	Contrast-enhanced CT of the abdomen and pelvis was requested.
3–4 h	The patient developed posterior left calf pain, progressive limb discoloration, and diminished distal Doppler signals. CT result—fissured left common iliac artery aneurysm with active contrast extravasation and a large retroperitoneal hematoma causing secondary ureteral compression and hydronephrosis. Vascular surgery team consulted.
After admission	Doppler ultrasonography and diagnostic angiography confirmed impaired lower-limb arterial flow. Urgent endovascular treatment with PTA and Bentley stent placement. Follow-up CT showed no further contrast extravasation. The patient was discharged after seven days without reported complications.

**Table 3:** Differential diagnosis of renal colic and retroperitoneal vascular emergencies in the ED [16,19,37,38,39,40].

Clinical Feature	Renal Colic	Retroperitoneal Vascular Emergency
Typical patient profile	May occur at any adult age; previous nephrolithiasis may be present	More concerning in older patients with hypertension, smoking, atherosclerosis, or known aneurysmal disease
Pain onset and character	Sudden, severe, often colicky or fluctuating pain	Frequently sudden and severe; may be described as tearing, stabbing, or persistent
Pain distribution	Flank pain commonly radiating toward the groin or testicle	Abdominal, flank, lumbar, groin, or referred testicular pain; distribution may change as bleeding or compression evolves
General appearance	Patient may be restless because of pain but is often otherwise clinically stable	Ill appearance, pallor, diaphoresis, or unexplained clinical deterioration may occur despite initially preserved blood pressure
Hematuria	Microscopic or macroscopic hematuria may support the diagnosis, although it can be absent	Often present in ruptured aortic aneurysms
Hemoglobin level	Usually preserved in uncomplicated disease	Anemia or a decreasing hemoglobin level may suggest concealed bleeding
Hydronephrosis on ultrasound	Usually caused by intrinsic ureteral obstruction, commonly a calculus	May result from extrinsic ureteral compression by an aneurysm or retroperitoneal hematoma
Vascular and limb findings	Peripheral perfusion is generally unaffected	New limb pain, discoloration, diminished pulses, or reduced Doppler signals may indicate vascular compromise
POCUS interpretation	Hydronephrosis may support ureteral obstruction when consistent with the overall presentation	A normal abdominal aorta does not exclude iliac vascular pathology; retroperitoneal bleeding may not be visualized
Role of CT	Non-contrast CT is commonly used when confirmation of ureterolithiasis is required	Contrast-enhanced CT or CT angiography may be required when vascular pathology or active bleeding is suspected

## Data Availability

All data supporting this work are available upon request to the corresponding author.

## Abbreviations

The following abbreviations are used in this manuscript:

AAA	Abdominal aortic aneurysm
CT	Computed tomography
ED	Emergency department
ESVS	European Society for Vascular Surgery
HDL	High-density lipoprotein
hpf	High-power field
LDL	Low-density lipoprotein
POCUS	Point-of-care ultrasound
PTA	Percutaneous transluminal angioplasty
RBCs	Red blood cells
